# Amorphous Calcium Phosphate-Based Bioactive Polymeric Composites for Mineralized Tissue Regeneration

**DOI:** 10.6028/jres.108.017

**Published:** 2003-06-01

**Authors:** D. Skrtic, J. M. Antonucci, E. D. Eanes

**Affiliations:** Paffenbarger Research Center, American Dental Association Health Foundation; National Institute of Standards and Technology, Gaithersburg, MD 20899-0001

**Keywords:** adhesion, amorphous calcium phosphate, dental composites, hydroxyapatite, mechanical strength, methacrylate conversion, tooth remineralization, volumetric contraction, water sorption

## Abstract

Amorphous calcium phosphate (ACP), a postulated precursor in the formation of biological hydroxyapatite, has been evaluated as a filler phase in bioactive polymeric composites that utilize dental monomers to form the matrix phase on polymerization. In addition to excellent biocompatibility, these composites provided sustained release of calcium and phosphate ions into simulated saliva milieus. In an effort to enhance the physicochemical and mechanical properties and extend the utility of remineralizing ACP composites to a greater variety of dental applications, we have focused on: a) hybridizing ACP by introducing silica and/or zirconia, b) assessing the efficacy of potential coupling agents, c) investigating the effects of chemical structure and compositional variation of the resin matrices on the mechanical strength and ion-releasing properties of the composites, and d) improving the intrinsic adhesiveness of composites by using bifunctional monomers with an affinity for tooth structure in resin formulations. Si- and Zr-modified ACPs along with several monomer systems are found useful in formulating composites with improved mechanical and remineralizing properties. Structure-property studies have proven helpful in advancing our understanding of the remineralizing behavior of these bioactive composites. It is expected that this knowledge base will direct future research and lead to clinically valuable products, especially therapeutic materials appropriate for the healing or even regeneration of defective teeth and bone structures.

## 1. Introduction

Calcium phosphates (CaPs) are of special interest to oral biology, dentistry and medicine since they occur in normal skeletal tissues (enamel, dentin, bone) and in pathological (atheroschlerotic deposits, urinary and dental calculi) calcifications [1]. Systematic studies of their use as preventive or restorative dental materials only began in the 1980s [2]. CaPs of biological significance are listed in Table 1. Crystalline HAP is considered to be the final, stable product in the precipitation of calcium and phosphate ions from neutral or basic solutions. Over the broad range of solution conditions in which precipitation occurs spontaneously, ACP precedes the appearance of HAP [3]. The possible role that ACP may play as a precursor to HAP in biological calcification places it in the mainstream of calcium phosphate chemistry [3–6]. In material applications, however, the relatively high solubility of ACP and its ready conversion to HAP in aqueous environments might pose limitations where structural, mechanical and chemical stabilities are desired. However, these same properties may make ACP suitable as a mineralizing agent. When compounded with appropriate polymeric resins, ACP’s bioactivity may be particularly useful in enhancing the prophylactic performance of composites, sealants and adhesives by preventing tooth demineralization and by actively promoting remineralization.

We have recently developed unique bioactive composites based on filler phases consisting of pyrophosphate-stabilized ACP embedded in certain types of polymer matrix phases derived from the ambient polymerization of acrylic monomers [7, 8]. Significant levels of calcium and phosphate ions were found to be released from these composites that were sustainable over long periods. It was found that they efficiently promoted the recovery of mineral-deficient tooth structures *in vitro* [9]. However, ACP does not act as a reinforcing filler in a manner similar to that of commonly used silanized glass fillers (Table 2). We are currently exploring protocols for hybridizing and surface-modifying ACP fillers and compounding hybrid and/or surface-modified ACPs with resins of different chemical compositions and structural characteristics in an effort to make stronger ACP composites. In this article we report on the effect(s) of the ACP filler type and/or the resin matrix structure on: 1) ACP’s stability and the rate of internal conversion to HAP, 2) visible light-polymerization efficacy, 3) volumetric contraction upon polymerization, 4) remineralization potential and 5) mechanical strength of the composites. Our current research is designed to lead to improved, remineralizing bioactive and biocompatible ACP composites with extended dental and potentially orthopedic applications.

## 2. Experimental

### 2.1 Formulation of Methacrylate Resins

**Fig. 1:**
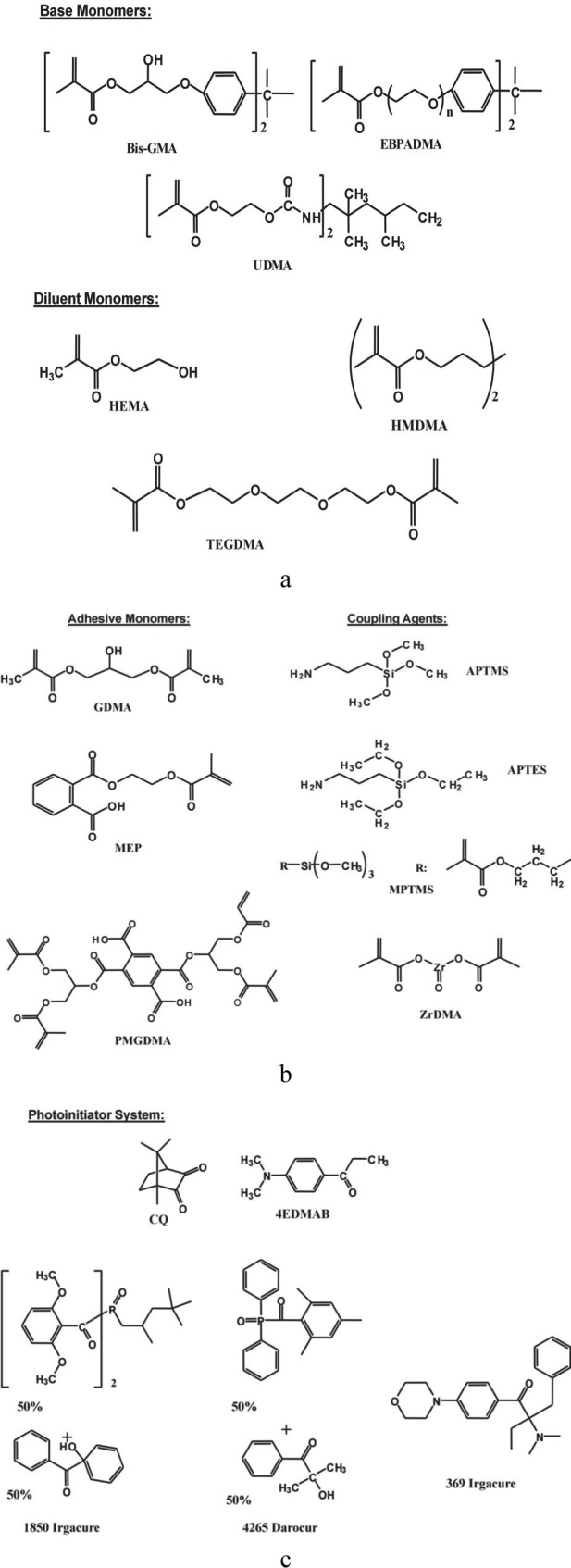
Chemical structure of the base and diluent monomers (a), adhesive monomers and coupling agents (b) and components of photoinitiator systems (c) used in the study.

The matrix resins were formulated from the commercially available dental monomers, coupling agents and components of the photoinitiator systems (Figs. 1a–c, Tables 3 and 4). Acronyms indicated in Tables 3 and 4 will be used throughout this manuscript. Bis-GMA-, EBPADMA- and UDMA-based resins were generally photoactivated by the inclusion of CQ and 4EDMAB as the photo-oxidant and photo-reductant, respectively. In the ETHM series, 1850 IRGACURE was utilized as the photoinitiator and for the PT resin a photoinitiator system consisting of 369 IRGACURE, 4265 DAROCUR and CQ was selected to enhance photopolymerization and storage stabiliy.

### 2.2 Synthesis and Characterization of ACP Fillers

**Fig. 2:**
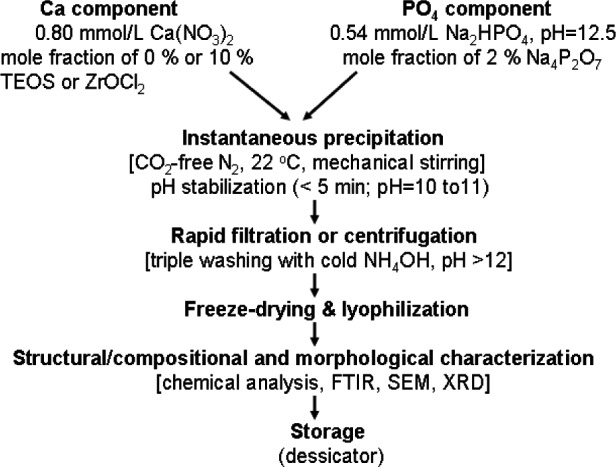
Schema of the experimental steps employed in the syntheses of ACP fillers.

The types of ACPs employed in the study are given in Table 5. Corresponding acronyms will be used in the manuscript hereafter. Syntheses of unmodified and hybrid ACPs were carried out according to a modified version of the preparation protocol proposed by Eanes et al. [10] (Fig. 2). Si-ACP was surface-treated as follows: APTMS, APTES or MPTMS was mixed into a slurry of ACP powder in cyclohexane containing a mass fraction of 2 % of n-propylamine (based on ACP). The suspension was then rotary evaporated (100 °C, moderate vacuum—2.7 kPa) to remove the solvents, cooled to room temperature (23 °C), washed with cyclohexane to remove residual silane and unattached products and re-dried under vacuum. ZrDMA was applied to Zr-ACP in a similar fashion from a methylene chloride solution.

The amorphous state of ACPs was verified by powder x-ray diffraction (XRD: Rigaku X-ray diffractometer^1^, Rigaku/USA Inc., Danvers, MA, USA) and Fourier-transform spectroscopy (FTIR: Nicolet Magna-IR FTIR System 550 spectrophotometer, Nicolet Instrument Corporation, Madison, WI, USA). The standard uncertainty of measuring the *d*-spacing values was 0.0013, and the measured *d*-values were within 0.05 % of the reported values of NIST SRM 640 (silicon powder, 2θ = 28.442, *d* = 3.1355). The wavelength accuracy of FTIR measurements was ≤ 0.01 cm^−1^ at 2000 cm^−1^. The particle size distribution (PSD) of the solids dispersed in isopropanol was determined by gravitational and centrifugal sedimentation analysis (SA-CP3 particle size analyzer, Shimadzu Scientific Instruments, Inc., Columbia, MD, USA) following 10 min ultrasonication. The Ca/PO_4_ ratio of the solids after dissolution in HCl was calculated from solution Ca^2+^ (atomic adsorption spectroscopy (AAS), Perkin Elmer Mo. 603 spectrophotometer (Perkin Elmer, Norwalk, CT, USA) and PO_4_ (UV/VIS Carey Model 219 spectrophotometer (Varian Analytical Instruments, Palo Alto, CA, USA, [11]) values. Additionally, AAS was employed to determine the amount of Si and/or Zr incorporated into hybrid fillers. Surface morphology of the fillers, after specimens were sputter-coated with gold, was determined by scanning electron microscopy (SEM: JSM-5400 instrument JEOL Inc., Peabody, MA, USA).

### 2.3 Physicochemical Evaluation of Resins, Pastes, and ACP Composites

**Fig. 3:**
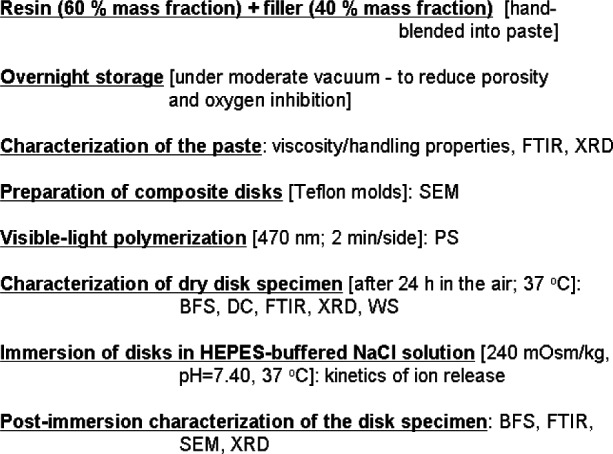
Schematic presentation of the experimental protocols utilized in physicochemical and mechanical assessment of ACP composites. Acronyms defined in text and Table 6.

The methodologies and techniques utilized to characterize and evaluate the methacrylate resins, the ACP fillers, and their composites are summarized in Table 6. The sequence of experimental steps employed in the physicochemical and mechanical evaluation of these bioactive ACP dental composites is schematically presented in Fig. 3. Composite pastes made up of various resins (Table 4; mass fraction 60 %) and ACP fillers (Table 5; mass fraction 40 %) were formulated by hand spatulation. The homogenized pastes were kept under a moderate vacuum (2.7 kPa) overnight to eliminate the air entrained during mixing. The pastes were molded into disks (15. 8 mm to 19.8 mm in diameter and 1.55 mm to 1.81 mm in thickness) by filling the circular openings of flat Teflon molds, covering each side of the mold with a Mylar film plus a glass slide, and then clamping the assembly together with a spring clip. The disks were photo-polymerized by irradiating sequentially each face of the mold assembly for 120 s with visible light (Triad 2000, Dentsply International, York, PA, US). After post-curing at 37 °C in air overnight, the disks were examined intact by XRD.

**Fig. 4:**
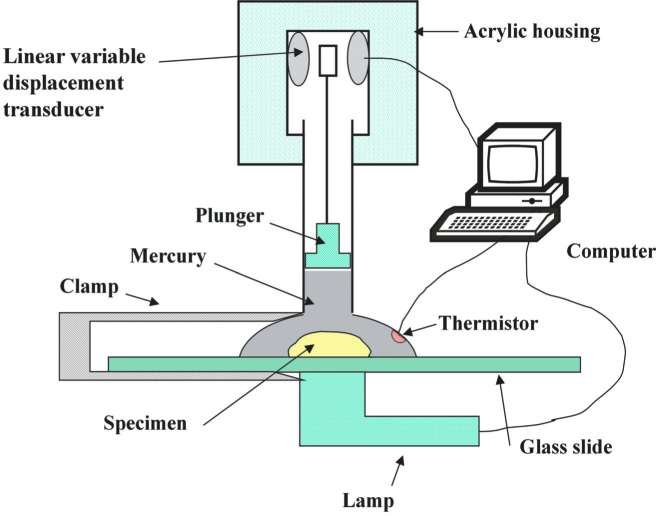
Schematic diagram of a computer-controlled mercury dilatometer used to determine volumetric contraction of the composites.

Volumetric contraction upon polymerization or polymerization shrinkage (PS) of the composites was measured by a computer-controlled mercury dilatometry (Fig. 4, [12]) that records the volume changes of the composite specimen, corrected for temperature fluctuations during the measurement, as a function of time and calculates the overall PS (volume fraction, %) based on the known mass of the sample and its density. Sample density was determined by means of the Archimedean principle using a water bath attachment to a microbalance (Sartorius YDK01 Density Determination Kit; Sartorius AG, Goettingen, Germany).

To determine the degree of methacrylate conversion (*DC*) attained after polymerization of the composites, a recently developed, non-destructive near infrared (NIR) spectroscopic technique for measuring the methacrylate conversion in dental resins was employed [13]. The absorption band at 6165 cm^−1^ in the overtone region was used to assess the *DC* in paired unpolymerized and polymer samples of known thickness. *DC* was calculated from the decrease in integrated peak area/sample thickness values in going from the unpolymerized to polymerized composites using the following expression:

$$DC=100\times \left[1-\frac{{\left(\text{area}/\text{thickness}\right)}_{\text{polymer}}}{{\left(\text{area}/\text{thickness}\right)}_{\text{monomer}}}\right].$$ (1)

The biaxial flexure strength (BFS) of each composite disk specimen was determined by using a computer-controlled Universal Testing Machine (Instron 5500R, Instron Corp., Canton, MA, US) operated by Instron Merlin Software Series 9. Detailed description of experimental protocols and calculations used in BFS screening are given in Ref. [14].

Mineral ion release from each individual composite disk specimen in a continuously stirred, HEPES-buffered (pH = 7.40) 240 mOsm/kg saline solution, was examined at 37 °C. Ca^2+^ and PO_4_ levels were determined by AAS and UV/VIS spectroscopy, respectively. Ion-release data were corrected for variations in the total area of disk surface exposed to the immersion solution using the simple relation for a given surface area, *A*: normalized value = (measured value) × (500/*A*).

To determine the water sorption (*WS*) profiles, a minimum of five replicate disks in each experimental group were initially dried over CaSO_4_ until a constant mass was achieved (± 0.1 mg). Specimens were then exposed to 75 % relative humidity (RH) at room temperature (23 °C) by keeping them over aqueous NaCl slurry in a closed system. Gravimetric mass changes were recorded at predetermined time intervals. Degree of water sorption (*WS*) of any individual specimen at any given time interval (*t*), expressed as a % mass fraction, was calculated using a simple equation:

$$WS=\left[\left(W_{t}–W_{0}\right)/W_{0}\right]\times 100,$$ (2)

where *W_t_* represents sample mass at the time *t*, and *W*_0_ is the initial mass of a dry sample.

One standard deviation was given in this paper for comparative purposes as the estimated standard uncertainty of the measurements. These values should not be compared with data obtained in other laboratories under different conditions.

## 3. Results and Discussion

**Fig. 5:**
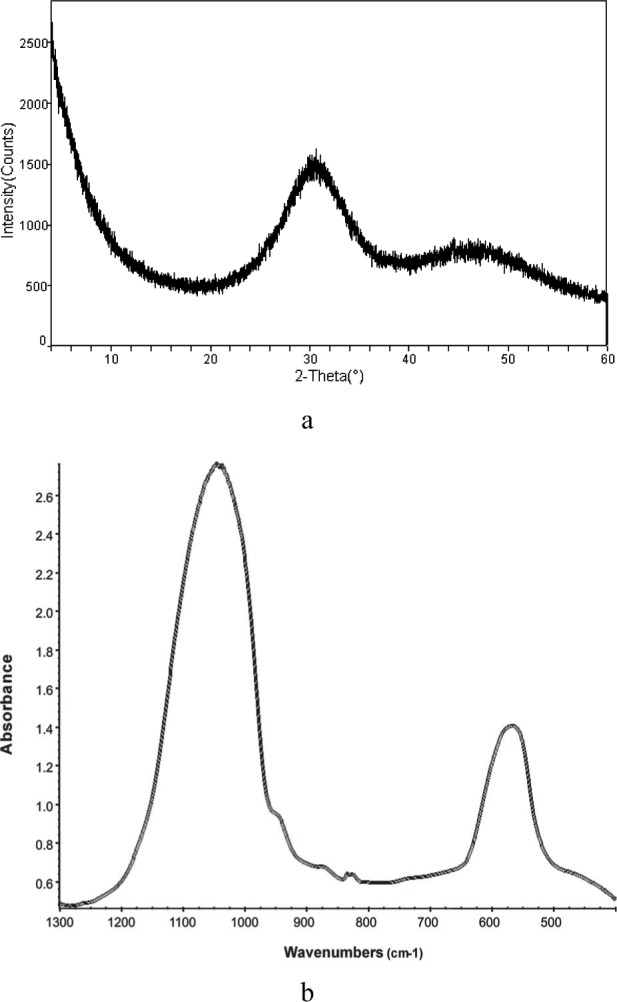
XRD pattern (a) and FTIR spectrum (b) of a representative ACP filler.

**Fig. 6:**
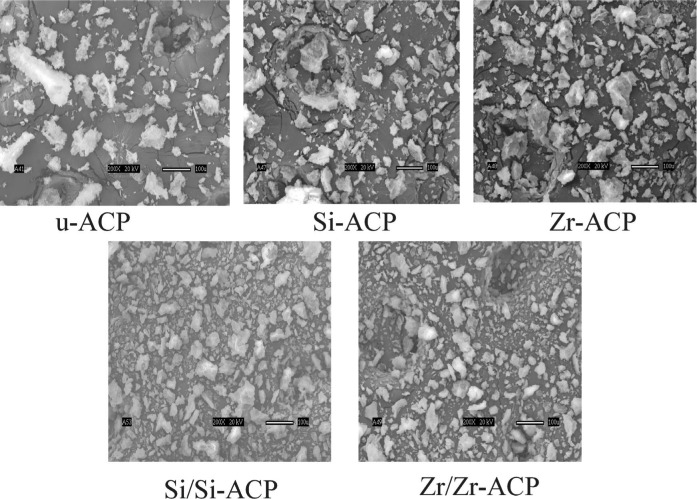
SEM microphotographs of unmodified, hybrid and surface-treated ACP powders.

**Fig. 7:**
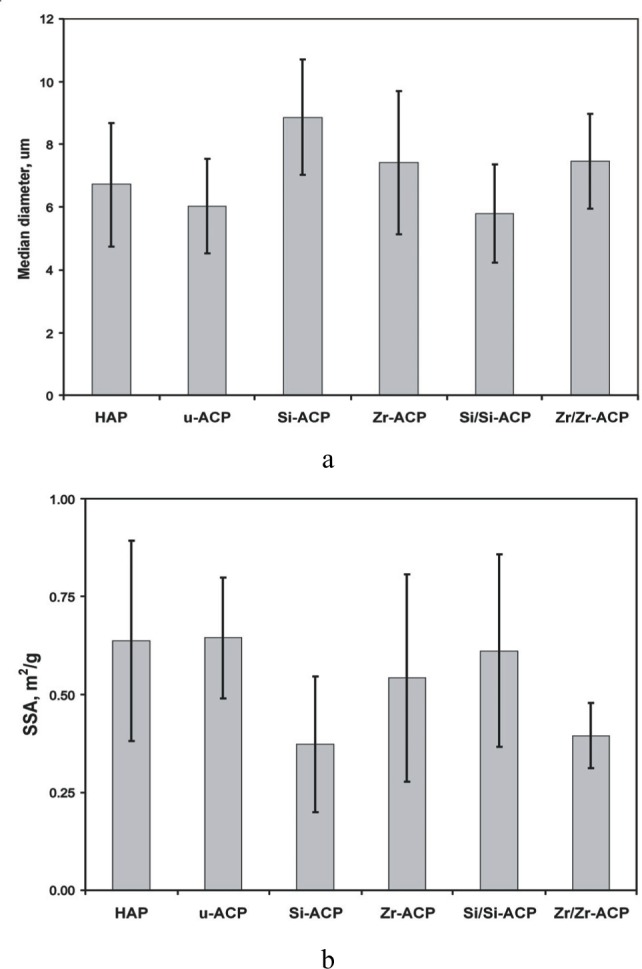
Mean values and standard deviations (indicated by bars) of the median particle diameter *d*_m_, (part a) and the corresponding SSA (part b) of various ACP fillers in comparison with HAP control. The standard deviation is taken as a measure of the standard uncertainty.

XRD patterns, FTIR spectra (representative scans are given in Fig. 5 a, b, respectively) and SEM images (Fig. 6) revealed no significant difference in structural and morphological features of unmodified, hybridized and surface-treated ACPs. All ACPs had heterogeneous PSDs with particle diameters (expressed as the equivalent spherical diameter) spanning from 0.1 µm to 80 µm. Apparent differences in the mean values of median diameters (*d*_m_; Fig. 7a) and the specific surface area (SSA; Fig. 7b) calculated from the corresponding PSDs of the powders were found to be greater than expected by chance (one-way ANOVA; *P* = 0.024). However, all pair-wise multiple comparisons (Tukey test) revealed that only the differences in *d*_m_ and SSA between the control HAP powder and Si-hybridized ACP are of statistical significance (*P* = 0.014 and *P* = 0.011, respectively). The observed increase in the *d*_m_, and consequently the lower SSA of Si-ACP compared to HAP, may be explained by different degree (extent) of agglomeration of the Si-hybridized filler. While the Ca/PO_4_ molar ratio of u-ACP and Si-treated ACPs was practically unchanged, it was significantly higher (Tukey test) for all Zr-treated fillers (Table 7). A speculative explanation for the observed compositional difference is that the loss of PO_4_ was caused by the formation of soluble Zr-PO_4_ complexes that paralleled the ACP precipitation, and that these soluble complexes were removed during the later stages of Zr-ACP and/or Zr/Zr-ACP synthesis. A lower level of incorporated Si compared to Zr in hybrid ACPs suggests that TEOS was most likely bound on the surface of the particles and, therefore, was more easily removed during filtering and washing of the freshly precipitated solids.

**Fig. 8:**
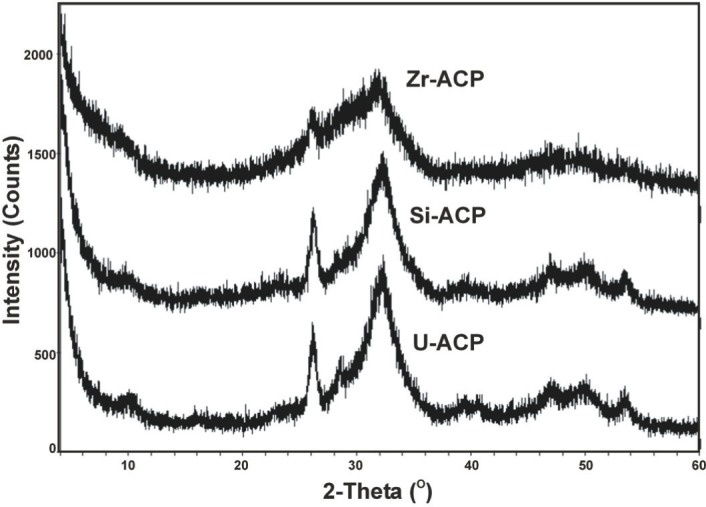
Stability of unhybridized (u-ACP) and hybrid ACPs (Si-ACP and Zr-ACP) in a test solutions containing 0.1 mol/L lactic acid and a mass fraction of 1 % hydroxymethyl cellulose (pH adjusted to 5.10 by NaOH solution). Time interval: 150 min. XRD patterns of Zr-ACP and Si-ACP show lesser conversion to hydroxyapatite than u-ACP (crystalline peaks marked by asterisk).

As evidenced by solution analysis, and by XRD and FTIR, the stability of ACPs exposed to different test solutions [15] decreased in the following order: Zr-ACP > Si-ACP > u-ACP (Fig. 8). Zr and Si retard the conversion of hybrid ACPs to HAP by their adsorption at HAP nucleation/growth sites. Since slower internal conversion into HAP is desirable in composites with remineralizing applications, these hybrid ACPs, especially Zr-ACPs, would appear to be the more suitable choice for use in bioactive dental materials.

**Fig. 9:**
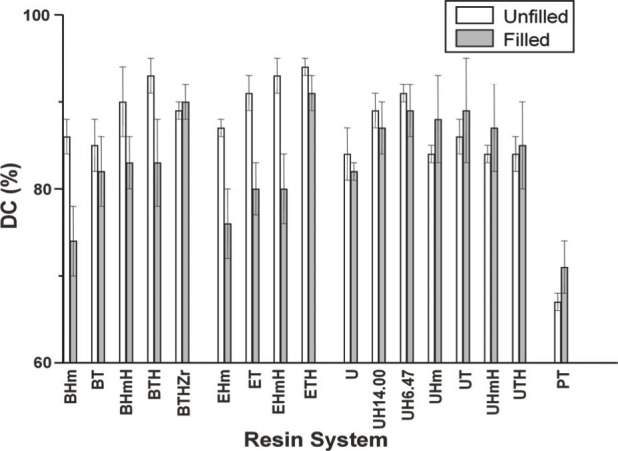
Mean values and standard deviations (indicated by bars) of the degree of methacrylate conversion, *DC*, of unfilled resins and corresponding ACP composites (average for u-ACP, Si-ACP and Zr-ACP fillers). Number of runs in each experimental group *n* ≥ 8. The standard deviation is taken as a measure of the standard uncertainty.

**Fig. 10:**
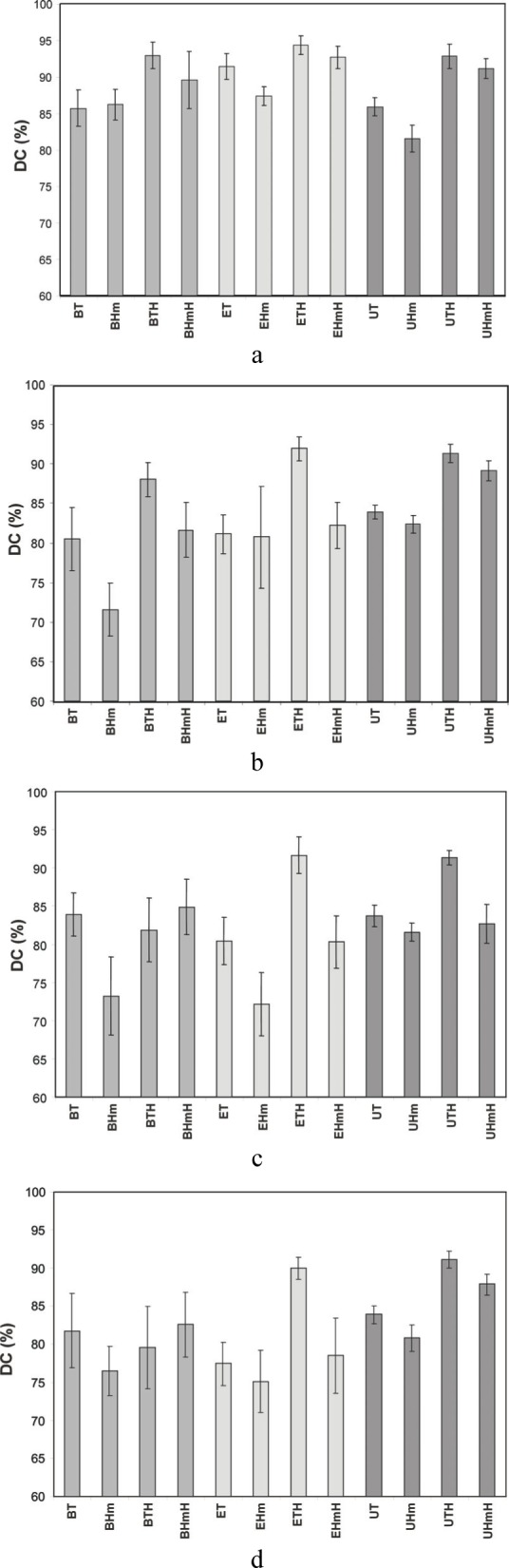
*DC* (mean value ± standard deviation (indicated by bars) of unfilled resins (a), u-ACP (b), Si-ACP (c) and Zr-ACP (d) composites as a function of resin composition. The standard deviation is taken as a measure of the standard uncertainty.

The results of the *DC* screening of experimental resins and ACP-composites are summarized in Fig. 9. Both unfilled Bis-GMA- and EBPADMA-based resins and to a lesser extent UDMA-based resins, as well as their ACP composites, achieved a higher methacrylate conversion when the hydrophilic, monofunctional HEMA was included as a co-monomer in the resin. Higher *DC*s for the resins with relatively high content of HEMA could be attributed to its high diffusivity and monofunctionality. High vinyl conversion, coupled with moderate contraction, was found in light-cured Bis-GMA resins containing hydroxypropyl methacrylate as a comonomer [16], a monomer homologous to HEMA. Regardless of the resin matrix composition the *DC*s of ACP-containing Bis-GMA- and EBPADMA-based composites were lower than UDMA-based composites. The UDMA monomer has already been been shown to be more reactive than Bis-GMA or EBPADMA [17]. No clear-cut trend could be established on the effect of filler type (u-, Si- or Zr-ACP) on the *DC* of the resin, except perhaps with regard to some of the composites formulated with HMDMA, e.g., BHm and EHm resins, which showed rather low *DC* with all ACP fillers. However, the following order of decreasing *DC*, independent of the filler type, can clearly be seen when the *DC* data are compared as shown in Figs. 10 a-d: XTH ≥ XHmH > XT ≥ XHm, with X being Bis-GMA, EBPADMA or UDMA. Furthermore, TEGDMA-containing matrices showed higher conversion that the corresponding HmDMA-containing ones (Student two-tail *t*-test; 95 % confidence interval). In conclusion, (Bis-GMA, EBPADMA or UDMA)/TEGDMA/HEMA formulations have the lowest potential for leaching out unreacted monomeric species. The significantly lower *DC* obtained with PT composites (most probably caused by the rigid aromatic core structure with practically no side-chain flexibility of PMGDMA) indicates a greater probability that the ACP-PT composites will release un-reacted monomers into the oral environment and consequently more likely will have a lower biocompatibility than Bis-GMA-, EBPADMA- and/or UDMA-based ACP composites.

**Fig. 11:**
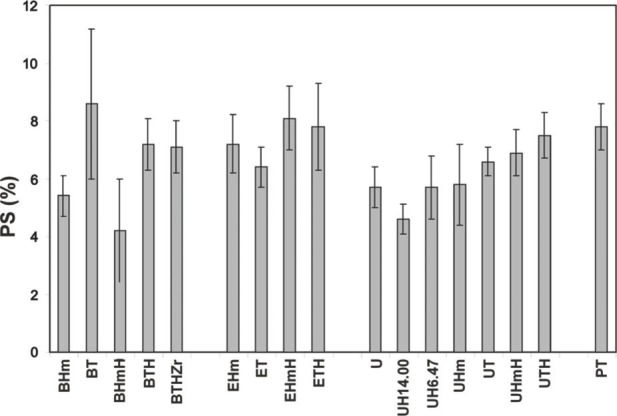
Mean values and standard deviations (indicated by bars) of the polymerization shrinkage of Bis-GMA-, EBPADMA- or UDMA-based ACP-filled composites (filler loading: 40 % mass fraction; average for u-ACP, Si-ACP and Zr-ACP) compared to TP composites. Number of runs in each experimental group *n* ≥ 9. The standard deviation is taken as a measure of the standard uncertainty.

The PS results showed a very complex dependence on both the resin composition and the filler type (Fig. 11). The majority of the experimental composites shrank more than did the commercial composite materials (*PS* 1.9 % to 4.1 % [18,19]), most probably due to the lower filler load (mass fraction of only 40 % ACP compared to that of (77 to 85) % of silica-based fillers in highly-filled conventional composites) and to ACP's heterogeneous size distribution. The experimental *PS* fell into the category of either flowable composites or adhesive resins ((3.6 to 6.0) % and (6.7 to 13.5) %, respectively [18]). Reformulated ACP-filled experimental composites should be further studied to determine if additional adjustments in resin formulations (bulkier but relatively low viscosity resins or ringopening monomers as the resin matrix component [20,21] might lead to composites with lower *PS* and optimal DMC.

Ion release from composites was affected by both the chemical structure and the composition of the monomer system as well as by the type of ACP filler (Table 8). Elevated Ca and PO_4_ concentrations were sustained in all but PT composites, which with increased time failed to maintain a favorable remineralizing potential due to the matrix uptake of released Ca *via* ion binding by the high concentration of carboxylic acid groups of PMGDMA [22]. Generally, the remineralizing capacity of ACP composites may be enhanced by a) introducing EBPADMA as a base monomer, b) elevating the level of HEMA in the resin formulation and c) by utilizing hybrid ACPs instead of u-ACP. The most probable mechanism by which the hydrophilic HEMA-enriched resins increased internal mineral saturation was by allowing the uptake of more water and/or better accessibility of hybrid ACP to the water already entrained. On the other hand, higher releases obtained with EBPADMA-based composites may partly be due to a more open cross-linked network structure of their resin matrix.

Results of the *BFS* testing of dry (before immersion) and wet (after immersion in saline solutions) composites are presented in Table 9 a-d. The mechanical strength of unfilled Bis-GMA- and EBPADMA-based resins did not deteriorate upon soaking. Unfilled UDMA-based and PT resins, however, failed to maintain their strength upon exposure to an aqueous environment. Generally, dry ACP-filled composites had substantially lower *BFS* than unfilled specimens regardless of the type of filler or resin matrix. The strength of all BisGMA-based (except the BTHZr resin), UDMA- based and PT composites deteriorated further upon soaking. Comparison of the *BFS* values of hybrid vs u- ACP/BTHZr composites showed modest but significant increase in the mechanical strength of Si- and Zr-ACP specimens. However, surface-treated Zr/Zr ACP composites failed to maintain their strength upon immersion. Recent m-FTIR mapping of ACP composites [23] indicated the existence of the numerous defects/voids (resin-rich, phosphate-depleted regions) in polymerized Si/MPTMS-ACP and Zr/Zr-ACP specimens compared to u- and hybrid ACPs. Uneven distribution of highly agglomerated, surface-treated ACP particulates throughout the matrix is, most probably, responsible for inadequate filler/resin interlocking and the resulting adverse effect on the overall mechanical strength. No clear-cut trend could be established for dry vs. wet EHm, ET, EHmH and ETH composites. However, the BFS of ETHM composites (formulated with MEP, proven to effectively promote bonding to dentin due to its surface activity [24]) decreased significantly upon soaking. The effect was independent of the resins' EBPADMA:TEGDMA ratio or the type of ACP filler utilized, except that again the weakening was more pronounced with surface-treated ACPs.

**Fig. 12:**
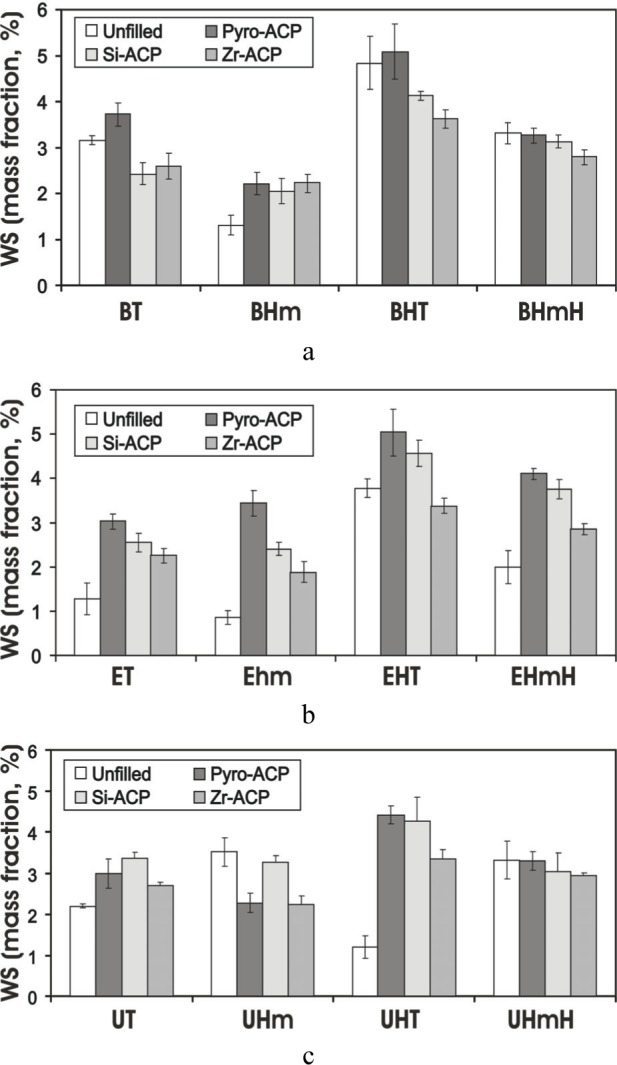
Maximum water sorption (*WS*) values [mean ± standard deviation (indicated by bars)] for unfilled (a) and ACP-filled XT, XHm, XTH and XHmH resins (X = Bis-GMA (part a) EBPADMA (part b) or UDMA (part c) T = TEGDMA, H = HEMA) after 5 weeks of exposure to 75 % relative humidity at 23 °C. Number of specimens in each group n ≥ 5. The standard deviation is taken as a measure of the standard uncertainty.

Studies conducted on the water sorption of dental materials indicate that excessive water uptake may cause a decrease in mechanical strength, distortion, and depression of the glass transition temperature [25] due to plasticization, solvation, reversible rupture of weak inter-chain bonds and irreversible disruption of the polymer matrix [26]. In the case of ACP/methacrylate composites, not only water-polymer but also water-ACP interactions occur and both contribute significantly to the overall water sorption profiles. Besides affecting the strength (*BFS* usually decreases upon soaking), water sorption/diffusion influences the mineral ion release kinetics and consequently the remineralizing ability of these bioactive materials. Kinetic WS data for unfilled and ACP-filled (u-, Si- and Zr-ACP) XT, XHm, XTH and XHmH (X = Bis-GMA, EBPADMA or UDMA, T = TEGDMA, H = HEMA) resins (data not shown) indicate that plateau values are reached within a week for XTH and within 2 weeks for XT, XHm and XHmH systems. Generally, XTH composites exhibited the highest and XHm composites exhibited the lowest *WS* (plateau values are compared in Fig. 12 a-c). Observed differences in *WS* are primarily due to whether hydrophilic (TEGDMA and HEMA) or hydrophobic (HmDMA) monomers are the dominant components of the resin matrices. No clear-cut conclusion could be made on the effect of the filler type on *WS*, although certain trends were evident: more water was absorbed by u-ACP composites compared to hybrid ACP composites in Bis-GMA and EBPADMA systems, Zr-ACP composites seemingly have the lowest *WS* regardless of the resin composition.

**Fig. 13:**
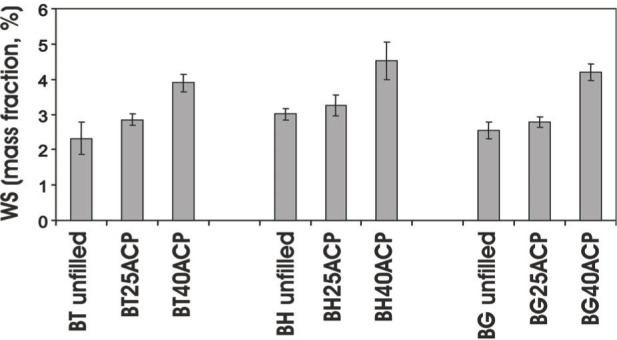
Maximum water sorption (*WS*) values [mean ± SD (indicated by bars)] of unfilled BT, BH and BG resins and corresponding composites with a mass fraction of 25 % or 40 % of Si-ACP after 5 weeks of exposure to 75 % relative humidity at 23 °C. Number of specimens in each group n ≥ 5. The standard deviation is taken as a measure of the standard uncertainty.

It has also been demonstrated that the filler's loading level has a significant effect on the *WS* of ACP composites [27]. As seen in Fig. 13, Si-ACP filled BT, BH and BG composites adsorbed more water than unfilled resin samples and the sorption was proportional to the mass fraction of Si-ACP in the composite. The differences may have resulted from increased stresses at the ACP/resin boundaries that, in turn, promoted water diffusion and enhanced the hydration of ACP surfaces. Also, the presence of silanol (≡Si-OH) groups in the Si-ACP could enhance *WS*. The possible relevance of this finding is that increased ACP levels increased *WS* which, in turn, increased ion release and faster ACP to HAP conversion. The resulting disruption in the integrity of the filler/resin interface decreased *BFS*.

These results exemplify the fact that water plays a very significant if not a major role in filler-matrix interactions. It may leach out filler elements, induce filler failures, cause filler-matrix de-bonding and reduce the strength of matrix material. Since the effect of water on most dental composites is irreversible [28], a true degeneration must have occurred either within the BT, BH or BG matrices or within TEOS/ACP “interphase” region of our experimental composites. Which region, the outer filler layer, the intra-silane coating or the silane-ACP bonds should be regarded as “critical” remains an open question. Through the future evaluation of hybrid ACP composites with reformulated resins, spectrum of their potential applications should extend to prevention of demineralization in orthodontically treated teeth and/or promotion remineralization of white spots in addition to initially envisioned applications as sealants and/or base/liner (Table 10).

## 4. Conclusions

In conclusion, results of this study demonstrate that it is possible to improve the remineralizing potential of ACP composites by introducing Si or Zr elements during the low-temperature synthesis of the filler. Si- and Zr- ACPs enhanced the duration of mineral ion release through their ability to slow down the intra-composite ACP to HAP conversion. Additionally, when compounded with BTHZr resins, hybrid ACPs showed improved mechanical properties compared to composites that utilized unmodified ACP. Also, the chemical structure and composition of the monomer system used to form the matrix phase significantly affected ion release, water sorption and the *DC* of the composites. Utilizing EBPADMA in addition to Bis-GMA as a base monomer and adding moderate amounts of hydrophilic HEMA may be the best route to maximize remineralizing ability of the filler while maintaining low leachability of unreacted monomeric species, i.e., high *DC*. However, additional adjustments in resin formulations will be necessary to improve the *PS* of current experimental ACP composites. Finally, Bis-GMA and EBPADMA have proven to be essential in maintaining the mechanical integrity of the composites. As currently formulated, resins that included surface-active adhesive monomers PMGDMA or GDMA did not meet basic physicochemical requirements for ACP composites.

## Figures and Tables

**Table 1:** Calcium phosphates (CaPs) of biomedical significance

CaP	Compositional formula	Acronym
Amorphous calcium phosphate	Ca_3_(PO_4_)_2_⋅3H_2_O^a^	ACP
Monocalcium phosphate	Ca(H_2_PO_4_)_2_	MCP
Dicalcium phosphate anhydrous	CaHPO_4_	DCPA
Dicalcium phosphate dihydrate	CaHPO_4_⋅2 H_2_O	DCPD
Tricalcium phosphate	Ca_3_(PO_4_)_2_	TCP
Octacalcium phosphate	Ca_8_H_2_(PO_4_)_6_⋅3H_2_O	OCP
Hydroxyapatite	Ca_10_(PO_4_)_2_(OH)_2_	HAP

a Approximate formula [3, 4].

**Table 2:** Functional differences between biostable and bioactive dental materials

Type of dental material	Components	Function/activity
Biostable restoratives	Monomer + initiator system	Provides polymeric matrix
	Silanized glass/ceramic filler	Reinforces matrix phase, enhances modulus and strength
Bioactive composites	Glass ionomers/resin modified ionomers/compomers	Release of fluoride ions from fluoride-containing filler
	Amorphous calcium phosphate (ACP) composites	Release of calcium and phosphate ions from ACP filler phase

**Table 3:** Monomers, coupling agents and photonitiator system employed in resin formulations

Component	Chemical nomenclature	Acronym
Base monomers	2,2-bis[p-(2′-hydroxy-3′-methacryloxypropoxy)phenyl]propane	Bis-GMA
	Ethoxylated bisphenol A dimethacrylate	EBPADMA
	Urethane dimethacrylate	UDMA
Diluent monomers	2-hydroxyethyl methacrylate	HEMA
	Hexamethylene dimethacrylate	HMDMA
	Triethyleneglycol dimethacrylate	TEGDMA
Adhesive monomers	Glycerol dimethacrylate	GDMA
	Methacryloyloxyethyl phtalate	MEP
	Pyromellitic glycerol dimethacrylate	PMGDMA
Coupling agents	3-aminopropyltriethoxysilane	APTES
	3-aminopropyltrimethoxysilane	APTMS
	methacryloxypropyl trimethoxysilane	MPTMS
	zirconyl dimethacrylate	ZrDMA
Photoinitiator system	Camphorquinone	CQ
	Ethyl-4-N,N-dimethylaminobenzoate	4EDMAB
	Bis(2,6-dimethoxybenzoyl)-2,4,4-trimethylpentylphosphine oxide & 1-hydroxycyclohexyl phenyl ketone	1850 IRGACURE
	diphenyl (2,4,6-trimethylbenzoyl) phosphine oxide & 2-hydroxy-2-methyl-1-phenyl-1-propanone	4265 DAROCUR
	2-benzyl-2-(dimethylamino)-1-(4-(4-morpholinyl)phenyl)-1-butanone	369 IRGACURE

**Table 4:** Composition (mass fraction %) of experimental resins evaluated in the study

a. Bis-GMA-based resins^a^								

Resin/monomer	Bis-GMA	HEMA	HMDMA	GDMA	PDMA	pHEMA	TEGDMA	ZrDMA
BG	55.00			44.00				
BH	68.35	30.65						
BHm	52.44		46.56					
BT	49.50						49.50	
BHmH	36.97	29.20	32.83					
BTH	35.50	28.00					35.50	
BTHZr	35.50	27.00					35.50	1.00

b. EBPADMA-based resins^b^					

Resin/monomer	EBPADMA	HEMA	HMDMA	MEP	TEGDMA
EHm	49.57		49.43		
ET	46.70				52.30
EHmH	34.33	30.44	34.23		
ETH	32.90	29.17			36.93
ETHM1.00^c^	42.00	10.00		5.00	42.00
ETHM0.67	33.60	10.00		5.00	50.40
ETHM0.50	28.00	10.00		5.00	56.00
ETHM0.25	16.80	10.00		5.00	67.20

c. UDMA-based resins^d^				

Resin/monomer	UDMA	HEMA	HMDMA	TEGDMA
U	99.00			
UH14.00^e^	92.40	6.60		
UH6.47	85.50	13.20		
UHm	51.75		47.25	
UT	48.82			50.18
UHmH	36.24	29.46	33.30	
UTH	34.83	28.32		35.85

d. Other^f^		

Resin/monomer	PMGDMA	TEGDMA
PT	48.65	48.65

a Photoinitiator system consisted of a mass fraction of 0.20 % CQ and 0.80 % 4EDMAB.

b Photoinitiator system consisted of a mass fraction of 0.20 % CQ and 0.80 % 4EDMAB except for the ETHM resins where a mass fraction of 1.00 % IRGACURE 1850 was utilized instead.

c Numbers represent a mass ratio EBPADMA: TEGDMA of 1.00, 0.67, 0.50 and 0.25, respectively.

d Photoinitiator system consisted of a mass fraction of 0.20 % CQ and 0.80 % 4EDMAB.

e Numbers represent a mass ratio UDMA: HEMA of 14.00 and 6.47, respectively.

F Photoinitiator system comprised a mass fraction of 0.40 % CQ, 0.80 % 4625 DAROCUR and 1.50 % 369 IRGACURE.

**Table 5:** ACP fillers employed in the study

Type of ACP filler	Stabilizing ion	Hybridizing agent	Surface-modifying agent	Acronym
Untreated ACP	P_2_O_7_^4−^	none	none	u-ACP
Hybridized ACP	P_2_O_7_^4−^	Tetraethoxy silane	none	Si-ACP
		(TEOS)^a^		
		Zirconyl chloride	none	Zr-ACP
		(ZrOCl_2_)^a^		
Surface- treated ACP	P_2_O_7_^4−^	TEOS	APTMS^b^	Si/APTMS-ACP
		TEOS	APTES^b^	Si/APTES-ACP
		TEOS	MPTMS^b^	Si/MPTMS-ACP
		ZrOCl_2_	ZrDMA^c^	Zr/Zr-ACP

a TEOS or ZrOCl_2_ were introduced *ab initio* during the ACP synthesis as a mole fraction of 10 % relative to calcium reactant. A mixture with mass fractions of 10 % TEOS, 10 % ethanol, 10 % tartaric acid and 70 % water, proven to effectively prevent premature TEOS gelation, was used to introduce the TEOS during hybridization.

b Si-ACP was surface-treated by introducing a mass fraction of 10 % of APTMS, APTES or MPTMS relative to ACP from a cyclohexane/n-propyl amine solution.

c Zr-ACP was surface-treated by introducing a mass fraction of 2 % ZrDMA relative to ACP from a methylene chloride solution.

**Table 6:** Methods and techniques employed in physicochemical characterization of the resins, fillers and ACP composites

Method	Property/parameter	Application/information
Atomic absorption spectroscopy (AAS)	Calcium, silica or zirconia concentration	Chemical analysis of ACP fillers Levels of hybridizing ions incorporated in ACP fillers Calcium release from composite disks exposed to aqueous environment
Computer-controlled mercury dilatometry	Volumetric contraction profiles	Volumetric polymerization shrinkage (PS) of composite resins upon light-polymerization
Fourier-transform infrared (FTIR) spectroscopy and microspectroscopy (m-FTIR)	Short-range structural arrangement	Structural/compositional properties of the monomers, ACP fillers, uncured and cured composites Degree of methacrylate conversion (DC) upon polymerization as indirect measure of the leachability of unreacted monomeric species Intra-composite ACP to HAP conversion
Gravimetry	Water sorption	Kinetics of the water uptake (water sorption, WS) by unfilled resins and ACP-filled composites
Mechanical testing	Physical strength	Biaxial flexure strength (BFS)
Particle size analysis	Cumulative and differential particle size distribution	Size (range, median diameter) and specific surface area of ACP fillers
Scanning electron microscopy (SEM)	Morphology, topology	Characterization of ACP fillers and composites before and after exposure to saliva-like solutions (soaking)
Ultraviolet/visible (UV/VIS) spectrophotometry	Phosphate concentration	Chemical analysis of ACP fillers Phosphate release from composite disks exposed to aqueous environment
X-ray diffraction (XRD)	Long-range crystalline order	Characterization of the fillers and uncured pastes Stability of ACP fillers upon immersion

**Table 7:** Elemental analysis of ACP fillers

Filler	Ca/PO_4_ molar ratio^a^	Incorporated hybridizing ion^b^ (mass fraction, %)
u-ACP	1.50 ± 0.09	
Si-ACP	1.59 ± 0.06	3.1 ± 0.5
Si/APTMS-ACP	1.56 ± 0.11	
Si/APTES-ACP	1.63 ± 0.15	
Si/MPTMS-ACP	1.49 ± 0.12	
Zr-ACP	1.91 ± 0.09	8.6 ± 1.4
Zr/Zr-ACP	2.19 ± 0.19	

All results are given as the mean values ± standard deviation.

a Number of repetitive experiments in each group *n* ≥ 5.

b Number of repetitive experiments in each group *n* = 5.

**Table 8:** Maximum concentration of the mineral ions (mean value for all types of ACP fillers) released from composites after 336 h of immersion in buffered saline. Number of runs in each experimental group n ≥ 9. *SD*s of the reported values ranged from (0.02 to 0.20) mmol/L and from (0.02 to 0.14) mmol/L for Ca and PO_4_ values, respectively

Resin	Ca (mmol/L)	PO_4_ mmol/L
BHm	1.33	0.60
BT	0.98	0.49
BHmH	1.52	0.68
BTH	1.29	0.58
BTHZr	0.97	0.62
EHm	2.52	0.62
ET	3.34	0.81
EHmH	4.73	1.17
ETH	3.13	0.80
PT	0.72	0.47
U	1.08	0.89
UH14.00	1.19	1.02
UH6.47	1.55	1.01
UHm	0.33	0.25
UT	1.06	0.64
UHmH	1.14	0.82
UTH	1.09	0.78

**Table 9:** Biaxial flexure strength (*BFS*) of dry (before immersion) and wet (after immersion in buffered saline for > 336 h) unfilled resins and ACP-filled composites. Results are indicated as mean value ± standard deviation with the number of specimens tested in each group given in parentheses

a. Bis-GMA-based resins			

Resin matrix		BFS (MPa)	
		Dry	Wet
BG	Unfilled copolymer	155 ± 32 (6)	131 ± 26 (6)
	composite	37 ± 4 (3)	17 ± 2 (4)
BH	Unfilled copolymer	167 ± 41 (6)	130 ± 27 (5)
	composite	42 ± 7 (4)	20 ± 6 (5)
BHm	Unfilled copolymer	101 ± 26 (4)	123 ± 26 (4)
	composite	53 ± 13 (12)	55 ± 11 (13)
BT	Unfilled copolymer	132 ± 27 (27)	123 ± 22 (17)
	composite	62 ± 15 (26)	62 ± 13 (26)
BHmH	Unfilled copolymer	155 ± 45 (4)	133 ± 36 (4)
	composite	71 ± 10 (12)	48 ± 8 (13)
BTH	Unfilled copolymer	156 ± 40 (8)	144 ± 52 (9)
	composite	56 ± 10 (28)	40 ± 9 (28)
BTHZr	Unfilled copolymer	116 ± 23 (25)	118 ± 30 (11)
	composite	69 ± 10 (118)	60 ± 14 (120)

b. EBPADMA-based resins			

		BFS (MPa)	
Resin	Filler	Dry	Wet
EHm	Unfilled copolymer	95 ± 18 (4)	93 ± 24 (4)
	composite	59 ± 8 (12)	53 ± 11(14)
ET	Unfilled copolymer	114 ± 19 (4)	125 ± 35 (4)
	composite	61 ± 6 (12)	59 ± 7 (15)
EHmH	Unfilled copolymer	122 ± 13 (3)	120 ± 27 (4)
	composite	58 ± 9 (12)	51 ± 9 (15)
ETH	Unfilled copolymer	133 ± 38 (3)	128 ± 49 (4)
	composite	57 ± 11 (12)	49 ± 8 (17)
ETHM1.00	Unfilled copolymer	110 ± 21 (4)	125 ± 8 (3)
	composite	69 ± 10 (29)	49 ± 8 (22)
ETHM0.67	Unfilled copolymer	128 ± 24 (4)	114 ± 27 (3)
	composite	72 ± 14 (14)	48 ± 9 (13)
ETHM0.50	Unfilled copolymer	130 ± 16 (4)	151 ± 38 (3)
	composite	76 ± 10 (17)	4 ± 6 (19)
ETHM0.25	Unfilled copolymer	135 ± 19 (4)	160 ± 37 (3)
	composite	65 ± 9 (19)	46 ± 10 (26)

c. UDMA-based resins			

		BFS (MPa)	
Resin	Filler	Dry	Wet
U	Unfilled copolymer	206 ± 15 (4)	159 ± 18 (4)
	composite	76 ± 6 (12)	53 ± 8 (12)
UH14.00	Unfilled copolymer	196 ± 6 (4)	155 ± 24 (4)
	composite	70 ± 8 (9)	61 ± 4 (12)
UH6.47	Unfilled copolymer	191 ± 29 (4)	151 ± 22 (4)
	composite	74 ± 3 (10)	53 ± 4 (12)
UHm	Unfilled copolymer	183 ± 22 (4)	117 ± 38 (3)
	composite	65 ± 8 (11)	60 ± 13 (14)
UT	Unfilled copolymer	192 ± 46 (4)	93 ± 30 (4)
	composite	61 ± 11 (12)	57 ± 10 (11)
UHmH	Unfilled copolymer	170 ± 32 (4)	123 ± 14 (4)
	composite	63 ± 7 (12)	37 ± 12(13)
UTH	Unfilled copolymer	124 ± 30 (4)	74 ± 27 (4)
	composite	54 ± 11 (12)	40 ± 11 (12)

d. Other			

		BFS (MPa)	
Resin	Filler	Dry	Wet
PT	Unfilled copolymer	164 ± 31 (5)	101 ± 29 (10)
	composite	72 ± 9 (40)	30 ± 6 (36)

**Table 10:** Potential benefits of bioactive ACP-based composites

Field	Foreseen benefit
Preventive dentistry	Remineralizing material that can counteract recurrent decay, known to develop near the surfaces of teeth in contact with conventional fillings (50 % of all dental fillings require replacement because of recurrent caries) • Particularly useful for patients that are especially susceptible to cavities as a result of radiation therapy and diseases or medications that cause dry mouth • Ameliorate the development and promote healing of root caries • Desensitizing agents for patients with tooth sensitivity
Orthodontics	Remineralizing adhesive cement that can minimize demineralization that frequently occurs under orthodontic brackets
Endodontics	Remineralizing root canal sealers or filling materials
Orthopedics	Biodegradable, potentially osteoconductive composites for healing defective bony tissues

## Footnotes

1 Certain commercial equipment, instruments, or materials are identified in this paper to foster understaning. Such identification does not imply recommendation or endorsement by the American Dental Association Health Foundation or the National Institute of Standards and Technology, nor does it imply that the materials or equipment identified are necessarily the best available for the purpose.
